# Cerebral Malaria and Neuronal Implications of *Plasmodium Falciparum* Infection: From Mechanisms to Advanced Models

**DOI:** 10.1002/advs.202202944

**Published:** 2022-10-27

**Authors:** Oscar Bate Akide Ndunge, Nicole Kilian, Mootaz M. Salman

**Affiliations:** ^1^ Department of Internal Medicine Section of Infectious Diseases Yale University School of Medicine 300 Cedar Street New Haven CT 06510 USA; ^2^ Centre for Infectious Diseases, Parasitology Heidelberg University Hospital Im Neuenheimer Feld 324 69120 Heidelberg Germany; ^3^ Department of Physiology Anatomy and Genetics University of Oxford Oxford OX1 3QU UK; ^4^ Kavli Institute for NanoScience Discovery University of Oxford Oxford UK; ^5^ Oxford Parkinson's Disease Centre University of Oxford Oxford UK

**Keywords:** 2D/3D in vitro models, 3D bioprinting, blood‐brain barrier, blood‐brain barrier‐on‐a‐chip, cytoadherence, endothelial cells, infectious diseases, malaria, neuron, *Plasmodium falciparum*, *Plasmodium falciparum* erythrocyte membrane protein 1, sequestration

## Abstract

Reorganization of host red blood cells by the malaria parasite *Plasmodium falciparum* enables their sequestration via attachment to the microvasculature. This artificially increases the dwelling time of the infected red blood cells within inner organs such as the brain, which can lead to cerebral malaria. Cerebral malaria is the deadliest complication patients infected with *P. falciparum* can experience and still remains a major public health concern despite effective antimalarial therapies. Here, the current understanding of the effect of *P. falciparum* cytoadherence and their secreted proteins on structural features of the human blood‐brain barrier and their involvement in the pathogenesis of cerebral malaria are highlighted. Advanced 2D and 3D in vitro models are further assessed to study this devastating interaction between parasite and host. A better understanding of the molecular mechanisms leading to neuronal and cognitive deficits in cerebral malaria will be pivotal in devising new strategies to treat and prevent blood‐brain barrier dysfunction and subsequent neurological damage in patients with cerebral malaria.

## Graphical abstract

Cerebral malaria is the deadliest complication patients infected with the protozoan parasite *Plasmodium falciparum* (*P. falciparum*) can experience. Herein the current understanding of the effect an infection with *P. falciparum* has on structural features of the human brain is detailed. Advanced 2D and 3D in vitro models are further assessed to study this devastating interaction between parasite and host.

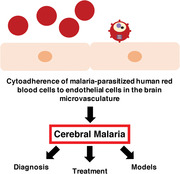

## 1 Malaria

Malaria is an ancient mosquito‐borne disease caused by protozoan parasites of the genus *Plasmodium*. According to the latest World Malaria Report published by the World Health Organization (WHO) in December 2021, 241 million clinical cases and 627 000 deaths were recorded in the previous year.^[^ ^1^, ^2^, ^3^ ^]^ Pregnant women and young children living in the WHO African Region (represented by the regional WHO office for Africa and comprised of 47 of the 54 African states) are the most affected by this devastating disease. *Plasmodium falciparum* (*P. falciparum*), one of the 6 human‐pathogenic *Plasmodium* species and the causative agent of malaria tropica, is responsible for the most clinical cases and deaths.^[^ ^4^ ^]^ The current situation in malaria endemic areas is unfortunately grim. The global COVID‐19 pandemic has further undermined, and will continue to undermine, years of progress in the fight against malaria.^[^ ^5^ ^]^ Identification of drug targets and vaccine candidates is therefore of great interest. The most promising vaccine candidate, RTS,S/AS01 (or Mosquirix), which was endorsed by the WHO in October 2021, is currently being tested in a pilot study roll‐out in Kenya, Ghana, and Malawi.^[^ ^6^ ^]^ It targets the circumsporozoite protein of the parasite to protect children against malaria.^[^ ^2^, ^3^, ^7^ ^]^ Other promising vaccine candidates are in earlier stages of clinical testing and development.^[^ ^8^, ^9^, ^10^ ^]^

### 1.1 The Life Cycle of *Plasmodium* Parasites

**Figure 1:**
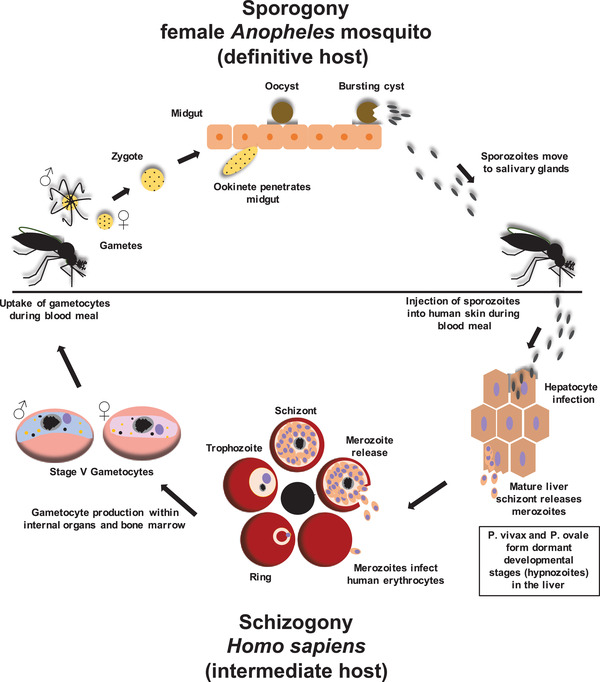
Life cycle of *Plasmodium spp*. The asexual reproduction, or schizogony, takes place in the intermediate host, while the sexual replication, or sporogony, happens in the definitive host. Throughout their life cycle, *Plasmodium* parasites can invade different cell types such as hepatocytes to initiate the liver schizogony and RBC to commence the erythrocytic schizogony. During the liver schizogony the sporozoite matures into a liver schizont that produces thousands of merozoites. These merozoites are released into the bloodstream where they infect the RBC of the human host for the first time and initiate the erythrocytic schizogony. Within the RBC the parasite develops into the so‐called ring stage. This is the only asexual developmental stage of the parasite that can be found in the peripheral blood of the patient. From the ring, the parasite develops into the trophozoite stage and finally into a merozoite‐producing schizont. The newly produced merozoites are released from the host RBC to find a new RBC for infection. The erythrocytic schizogony is responsible for the well‐known symptoms malaria patients experience. To facilitate the uptake by a female *Anopheles* mosquito, sexual stages (or gametocytes) must develop. Eventually, some parasites commence their 10‐day‐long sexual maturation. The development process of Gametocytes is divided into 5 morphological stages (I–V). In order to conduct the maturation process, immature gametocyte stages (I–IV) sequester in internal organs and the bone marrow. Only the mature stage male and female V gametocytes are released back into the bloodstream to serve their purpose of being taken up for mating by a female *Anopheles* mosquito during the blood meal. Within the midgut of the mosquito, male and female gametocytes mature into gametes and eventually mate by fusing into an ookinete, which migrates across the epithelial cells of the mosquito midgut. The ookinete settles beneath the epithelial cells of the midgut and develops into a sporozoite‐producing oocyst. Mature sporozoites then burst out of the oocyst and invade the salivary glands of the mosquito where they wait until for the next blood meal. During this blood meal, the sporozoites are then inoculated into the intermediate host where they first invade the hepatocytes of the liver in order to start their asexual replication. Reproduced under the terms of the Creative Commons CC‐BY license.^[^ ^12^ ^]^ Copyright 2019, The Authors. Published by Wiley‐VCH

*Plasmodium* parasites are defined by their complicated life cycle (**Figure** **1**). To execute this life cycle, the parasite must establish an infection in a human intermediate host and a mosquito of the genus *Anopheles* as definitive host. Life cycle progression and production of parasite progeny are ensured by the successful infection of various cell types.^[^ ^11^ ^]^

The blood meal of the female *Anopheles* mosquito commences the infection of the human intermediate host. Sporozoites invade and reside in the salivary glands of the mosquito and are inoculated into the skin of the human host together with the saliva during the blood meal. The sporozoites must travel from the bite site toward a blood vessel to reach the bloodstream, which will eventually carry them to the liver sinusoids. There the sporozoites exit the bloodstream again in order to infect hepatocytes.

Within hepatocytes, sporozoites develop into merozoite‐producing liver schizonts (liver schizogony). The haploid merozoites are then released into the bloodstream. Interestingly, the causative agents of malaria tertiana, *P. vivax*, and *P. ovale*, have the ability to form dormant and persisting developmental stages, so‐called hypnozoites, which remain within the liver and can cause malaria relapse years after the initial infection.

The erythrocytic schizogony begins once the liver merozoites start to infect the red blood cells (RBC) of the human host. During the erythrocytic schizogony the parasite matures from the juvenile ring stage via the trophozoite stage to the schizont stage. The parasite remains in the ring stage for ≈24 h. During this developmental stage, the parasite is highly motile within the RBC. One or more ring stages can be found within RBC of the peripheral blood of a patient infected with *P. falciparum*, which serves as diagnostic hallmark.^[^ ^13^ ^]^ Afterward, the parasite matures into a stationary and fast‐growing trophozoite. Finally, the parasite enters the schizont stage during which it generates up to 32 daughter merozoites which are released into the bloodstream after schizont rupture to continue the erythrocytic schizogony and invade new RBC. The erythrocytic schizogony of the parasite is responsible for the pathophysiology of malaria. Merozoite egress releases a multitude of pyrogens from the ruptured host RBC which cause the hallmark fever already described centuries ago. During the erythrocytic schizogony, some parasites eventually differentiate into sexual developmental stages, so‐called gametocytes or sexual precursor cells (female microgametocytes and male microgametocytes). These sexual precursor cells must be ingested by a female *Anopheles* mosquito during the blood meal to commence the sporogony. The gametocytes develop into gametes and mate to form a zygote within the midgut of the mosquito. This zygote develops into an ookinete that penetrates the midgut epithelial cells and develops into an oocyst. The oocyst produces sporozoites which are released after its rupture and invade the salivary glands of the mosquito. These sporozoites are then inoculated into the human intermediate host during the next blood meal of the mosquito.

### 1.2 Parasite‐Driven Red Blood Cell Reorganization and Host‐Parasite Interaction

**Figure 2:**
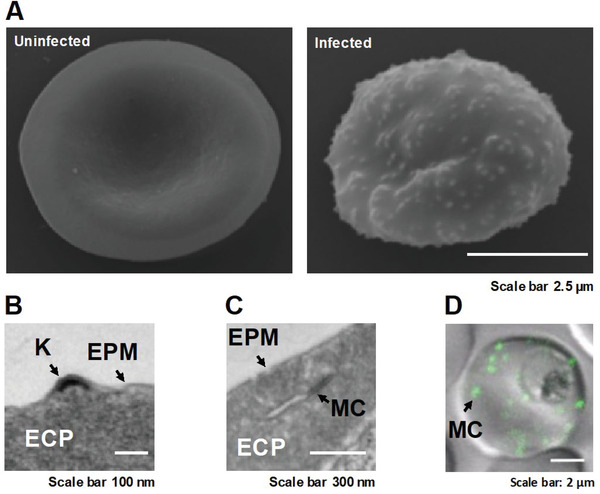
Reorganization of the host RBC by the malaria parasite *Plasmodium falciparum*. A) Scanning Electron Microscopy (SEM) images of an uninfected and an infected RBC show the marked changes exerted by the parasite. The RBC loses its biconcave shape while the parasite grows. Small protrusions, so‐called knobs, are presented on the surface of the infected RBC. The knobs serve as presentation platforms for the cytoadherent parasite protein PfEMP1. B) Transmission Electron Microscopy (TEM) image of a thin sectioned infected RBC shows the electron density of the knob. The successful establishment of knobs depends on the Maurer's clefts, a novel secretory organelle generated by the parasite in order to facilitate protein trafficking in the usually cell‐organelle‐deprived RBC C). The Maurer's clefts consist of several single cisternae or a few stacks of several cisternae which are usually spread throughout the infected RBC. The morphology of the organelle depends on the parasite strain. D). The confocal image shows the location of the Maurer's clefts via the GFP‐tagged PfSBP1 (*P. falciparum* skeleton binding protein 1) which attaches the cisternae of the newly generated Maurer's clefts to actin filaments that have been mined from the membrane skeleton of the RBC by the parasite. Reproduced under the terms of the Creative Commons CC‐BY license.^[^ ^12^, ^325^ ^]^ Copyright 2019, The Authors. Published by Wiley‐VCH.

Erythrocytic schizogony completion requires a significant reorganization of the host RBC cytoplasm and the RBC membrane to ensure the survival of the parasite by providing essential nutrients and protection against the immune system of the host ^[^ ^14^ ^]^ (**Figure** **2**). After parasite invasion, the RBC undergoes several morphological and rheological alterations, which are manifested by increased RBC membrane rigidity and reduced deformability.^[^ ^15^ ^]^ Reorganization of the host RBC eventually culminates in the generation of a novel secretory organelle of parasite origin, the Maurer's clefts, that resides within the host RBC cytoplasm (Figure 2C,D). During the ring stage of the parasite the Maurer's clefts are single vesicles which move throughout the RBC cytoplasm via Brownian motion.^[^ ^16^ ^]^ The vesicular structures of the Maurer's clefts eventually flatten to form single or stacked cisternal structures which are tethered in close proximity to the plasma membrane of the infected RBC (iRBC) via actin filaments.^[^ ^16^, ^17^ ^]^ The necessary actin monomers to form these important actin filaments are mined from the hexagonal actin‐spectrin membrane skeleton of the host RBC by the parasite.^[^ ^17^ ^]^ This actin‐facilitated tethering of the flattened Maurer's clefts cisternae is a necessary structural change in organelle morphology and behavior to allow the essential trafficking of proteins of parasite origin that occurs during the trophozoite stage of the parasite.^[^ ^18^ ^]^ The Maurer's clefts receive dozens of different proteins of parasite origin, and have multiple functions. However, arguably the most important function of the Maurer's clefts is the trafficking of essential parasite proteins to commence successful host‐parasite interaction.^[^ ^16^, ^17^, ^18^ ^]^ One of these essential proteins is PfEMP1 (*P. falciparum* erythrocyte membrane protein 1). The genome of *P. falciparum* encodes for 60 versions (*var* gene family) of this immunovariant adhesin, which is essential for host parasite interaction.^[^ ^19^ ^]^ Every parasite expresses only one PfEMP1 variant at a time, but is able to switch between variants.^[^ ^19^ ^]^ PfEMP1 is trafficked to the surface of the infected RBC and presented on protrusions, so‐called knobs, on the plasma membrane of the host cell ^[^ ^20^ ^]^ (Figure 2A,B).

The appropriate transport and presentation of PfEMP1 are crucial to establish cytoadherence, which allows the binding of the iRBC to specific receptors presented on different cells in the human body, such as syncytiotrophoblasts in the placenta (causing placental malaria) and endothelial cells (EC) that line the postcapillary venules within organs such as the brain, kidneys, and lungs. Receptors on the surface of the iRBC include PfEMP1, 2, and 3, histidine‐rich protein I and II, sequestrin, rosettins, and ring‐infected RBC membrane surface antigens (Pf155/RESA (*P. falciparum* 155/Ring‐Infected Erythrocyte Surface Antigen precursor)).

PfEMP1 is the most abundant protein on the surface of iRBC. PfEMP1 is able to bind one or two specific receptors present on the surface of the EC or syncytiotrophoblasts.^[^ ^19^ ^]^ EC present specific adhesion molecules for the regulation of cell adhesion and permeability. These EC include intercellular adhesion molecule‐1 (ICAM‐1), vascular cell adhesion molecule‐1 (VCAM‐1), cluster of differentiation 36 (CD36), E‐selectin, chondroitin sulfate A (which is also presented by syncytiotrophoblasts), thrombospondin, endothelial leukocyte adhesion molecule‐1^[^ ^21^, ^22^ ^]^ and the cytokine‐activated endothelial protein C receptor (EPCR).^[^ ^23^ ^]^

Binding between PfEMP1 and the matching receptor determines where the cytoadherence occurs and effectively retains the iRBC within the vascular bed of the organ by mimicking leukocyte rolling adhesion until the erythrocytic schizogony is completed and the merozoites have left the sequestered iRBC in order to find and infect a new host RBC.

Cytoadherence and sequestration of RBC infected with mature developmental stages (trophozoite and schizont) of *P. falciparum* are essential to avoid the passage of the spleen where infected and deformed RBCs are removed from blood circulation. The increased time that the iRBC remain within inner organs results in obstruction of microvascular blood flow, hypoxia, and ischemia.^[^ ^24^, ^25^, ^26^ ^]^ Cerebral malaria (CM) as a severe symptom can occur when iRBC, as well as platelets and microparticles, cytoadhere to the vascular endothelium in order to sequester in the brain of the patient.

### 1.3 Hemoglobinopathies, Nature's Way of Protection against Disease Severity

Disease severity and death as a result of infection with *P. falciparum* lead to a high selective pressure in malaria endemic areas. Many genetic disorders with stable polymorphisms have evolved in order to protect their carriers. Hemoglobinopathies, for example, have evolved as a direct response to the existing selective pressure which occurs in malaria endemic areas. The most commonly known protective hemoglobins are the sickle cell hemoglobin (HbS) and hemoglobin C (HbC).^[^ ^27^ ^]^ Both hemoglobins result from a single point mutation in the gene encoding for the *β‐*globin chain of the oxygen‐transporting hemoglobin molecule. This, in turn, leads to the substitution of a single amino acid at position 6 in the *β*‐globin chain with a glutamic acid residue replaced by either a valine or lysine residue in HbS and HbC, respectively.

In contrast, other hemoglobinopathies known as *α*‐ and *β*‐thalassemia are caused by aberrant splicing of mRNAs encoding the *α*‐ and *β*‐globin chains.^[^ ^28^, ^29^, ^30^ ^]^

How these hemoglobinopathies protect from severe malaria is only partly understood. Proposed mechanisms include reduced disease‐mediating cytoadherence of iRBC, impaired intraerythrocytic development of the parasite, dampened inflammatory responses, or a combination thereof which ultimately leads to the avoidance of CM and other hallmarks of disease severity.^[^ ^17^, ^31^, ^32^, ^33^ ^]^ Unfortunately, not every person exposed to *P. falciparum* is protected by their genes. Therefore, development of CM as a result of *P. falciparum* infection remains a serious threat.

## 2 Structural Features of the Human Brain

### 2.1 Biology of the Blood‐Brain Barrier

The blood‐brain barrier (BBB) separates the peripheral blood from the brain microenvironment and serves as strong protection against pathogens and neurotoxins.^[^ ^34^ ^]^ It is made up of a monolayer of highly specialized EC, so‐called brain microvascular endothelial cells (BMVECs), that line the vascular walls and are firmly held together by several selective tight and adherens junction proteins (Zona occludens‐1, ‐2, ‐3 (ZO‐1, ZO‐2, ZO‐3), occludins, claudins, cingulin), cadherin–catenin complex and the underlying basal lamina.^[^ ^35^, ^36^ ^]^ The specialized EC are embedded within an extracellular matrix and are surrounded by supporting cells called glial cells such as pericytes and astrocytes, along with other glial cells such as microglia and oligodendrocytes.^[^ ^37^, ^38^, ^39^ ^]^ The role of glial cells involves supporting neuron function through regulating neurotransmitter release, water, and ion homeostasis,^[^ ^40^, ^41^, ^42^ ^]^ glymphatic function,^[^ ^43^, ^44^ ^]^ brain energy and metabolism,^[^ ^45^ ^]^ in addition to synapse formation and maintenance.^[^ ^46^, ^47^ ^]^

A proper functioning BBB is important to ensure homeostatic control of the central nervous system (CNS) by responding to changes in water, ion, or molecule homeostasis as well as directing and checking the transport of nutrients such as glucose and amino acids from the blood to the CNS.^[^ ^39^, ^41^ ^]^ The BBB further has a crucial function in the removal of metabolic waste products from the CNS as well as regulating brain energy, regional oxygen, and nutrient levels.^[^ ^39^, ^48^ ^]^

Loss of BBB integrity results in dysregulated transport, neurotoxin infiltration, local and generalized immune response, and neuroinflammation and is known to be implicated in a wide range of CNS disorders including Alzheimer's disease, Parkinson's disease, amyotrophic lateral sclerosis (ALS), and multiple sclerosis (MS), as well as brain tumors.^[^ ^49^ ^]^

### 2.2 Brain Endothelial Cells and Their Vascular Functions

The brain endothelium is composed of a thin single sheet of specialized EC.^[^ ^50^, ^51^ ^]^ The ECs are enclosed by pericytes, basement membrane, and astrocytes end‐feet, establishing a neuro‐vascular unit.^[^ ^52^ ^]^ These astrocyte end‐feet encircle the blood vessel (arterioles), connecting the astrocytes to neurons, and neighboring oligodendroglia and microglia cells,^[^ ^53^, ^54^ ^]^ forming together tight junction (TJ) complexes which mediate the so‐called transcellular and paracellular pathways, and serve as a protective barrier for the brain parenchyma.^[^ ^55^, ^56^ ^]^ This metabolically active layer covers the inner surface of blood vessels and serves as an interface between blood and brain tissue, protecting the brain parenchyma from toxins, injury, inflammation, pathogens, and drug entry.^[^ ^39^, ^52^, ^57^ ^]^

Through secretion of vasodilators and vasoconstrictors, a variety of blood vessel functions including blood fluidity and passage of nutrients, hormones, and macromolecules to surrounding tissues are regulated.^[^ ^58^, ^59^ ^]^ The maintenance of vascular tone, cell adhesiveness, platelet aggregation, leukocyte trafficking, coagulation cascade, inflammation, permeability, regulation of thrombosis, and fibrinolysis are also major functions of the brain endothelium.^[^ ^39^ ^]^ In addition, some vasoprotective effects of a healthy endothelium include vasodilation and inhibition of inflammatory responses, which is done by maintaining a homeostasis between the vasodilators such as nitric oxide (NO), prostacyclin (PGI2), and endothelium‐derived hyperpolarizing factor as well as vasoconstrictors, such as endothelin‐1 (ET‐1), angiotensin II (Ang II), thromboxane A2 (TXA2) and platelet‐activating factor released by the EC.^[^ ^60^, ^61^, ^62^ ^]^ Activation of the vascular endothelium induces inflammation through upregulation of EC adhesion molecules such as vascular cell adhesion molecule‐1 (VCAM1), ICAM1, and E‐ and P‐selectins, which provokes leukocytes rolling and adherence to vascular endothelium, ultimately facilitating leukocytes transmigration and extravasation into the brain parenchyma.^[^ ^50^ ^]^ Once extravasated into the brain parenchyma, these peripheral immune cells initiate brain tissue damage by free‐radical reactions and NO depletion, which can cause vasoconstriction and ischemia.^[^ ^63^, ^64^ ^]^ Many physiological processes are promoted by NO. Under basal conditions, vascular ECs synthesize and release nitric oxide. Once synthesized and released, NO triggers vasodilation and exerts a protective effect by inhibiting platelet aggregation, decreasing immune cells' adhesion to the vascular endothelium, and increasing blood flow to affected brain regions.^[^ ^65^, ^66^ ^]^ NO also acts as a signaling molecule by stabilizing the endothelium via the anti‐inflammatory and anti‐coagulatory pathways through inhibition of angiopeoietin‐2 (Ang‐2) secretion from Weibel‐Palade Body (WPB) and induction of Ang‐1 secretion.^[^ ^36^ ^]^ So far, three isoforms of nitric oxide synthase (NOS) have been identified. Endothelial cell NOS (eNOS; NOS3) and neuronal NOS (nNOS; NOS1), are expressed constitutively while the inducible isoform (iNOS; NOS2) is expressed in response to inflammatory stimuli, such as parasitic/bacterial products, cytokines, and lipid mediators.^[^ ^67^ ^]^ Endothelium‐derived NO (eNOS) is expressed in vascular EC and regulates NO production by EC. It is believed that inflammatory cytokines increase inducible NO synthase in brain ECs, causing increased NO production. In fact, endothelium‐derived NO (eNO) plays an important pro‐ or anti‐inflammatory role in the regulation of NO production depending on pathophysiological conditions, timing, and location.^[^ ^67^ ^]^ Within the brain parenchyma, eNOS inhibition reduces BBB dysfunction. On the other hand, excess NO release directly increases BBB disruption because it is considered to be a “damaging” radical (ROS), which might cause oxidative/nitrosative tissue injury and potentially dysregulating neuroinflammation.^[^ ^66^, ^68^, ^69^, ^70^, ^71^ ^]^ Another important aspect of the ECs is their involvement in coagulation through von Willebrand Factor (vWF). vWF is found in endothelial storage granules, called Weibel–Palade bodies. Once secreted, vWF plays the role of protective protein of the coagulation factor VIII. During vascular injury, vWF is released and binds to the damaged vessel wall and platelets. This causes platelet aggregation and blood clotting within the injured area and prevents further leakage of blood into the surrounding brain tissue.^[^ ^72^ ^]^

## 3 The Effect of *P. falciparum* Infection on Brain Endothelial Cells

### 3.1 Microvascular Obstruction and Activation of Endothelial Cells

**Figure 3:**
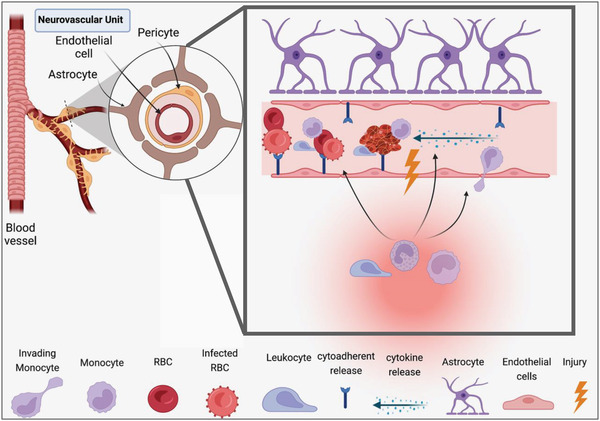
Pathophysiology of cerebral malaria. Cerebral malaria pathophysiology occurs after *P. falciparum* infection, endothelial cell dysfunction, blood‐brain barrier (BBB) disruption, and neuroinflammation. The BBB consists of three major cellular types which are the brain vascular specialized endothelial cells, astrocytes, and pericytes. The BBB endothelium has a limited transcellular transport and restricted transcellular transport in order ensure optimum environment for neuronal function and protect the CNS against the invasion of pathogens, toxins, proinflammatory cytokines, and chemokines. In CM, both parasite and the host immune response promote the release of proinflammatory molecules that can damage brain endothelial cells and impair the integrity of tight junction, leading to BBB leakiness and dysfunction. This would allow peripheral immune cells and circulating cytokines to enter the CNS causing neuroinflammation and resulting in cognitive deficits.

*Plasmodium* parasite infection induces the endothelium to adopt a vasoconstrictive, proinflammatory and prothrombotic state. Increased vasoconstriction, inflammation, and coagulation cause abnormal functioning of EC, resulting in ED. Perturbations in BBB integrity and the recruitment of peripheral immune cells are hallmarks of ED.^[^ ^73^, ^74^ ^]^ The cerebral endothelium plays a critical role in CM through cytoadherence of iRBC (trophozoites and schizonts stages) to the brain microvasculature, causing vasospasms and inducing changes in levels of vasoregulatory molecules, hypoperfusion and ischemia as well as inflammation and leakage of the BBB.^[^ ^75^, ^76^, ^77^, ^78^, ^79^, ^80^, ^81^, ^82^, ^83^, ^84^ ^]^ Cerebral blood circulation is obstructed by sequestered iRBC and platelets to the endothelium. Restriction of blood flow by both rigid iRBC and uninfected RBC as well as by clumping of iRBC with platelets, leukocytes and uninfected RBC (rosette formation) causes increased vasoconstriction, BBB disruption, and elevated inflammation ^[^ ^85^, ^86^, ^87^, ^88^, ^89^, ^90^ ^]^ (**Figure** **3**). Cytoadherence and sequestration of iRBC in the brain occur through ICAM‐1 and EPCR, two molecules which are expressed on the surface of brain ECs, and specific variants of PfEMP1 which are presented on the surface of iRBC.^[^ ^19^, ^91^, ^92^ ^]^ Binding between ICAM‐1 and EPCR on EC causes clustering of ICAM‐1 receptors on the cell surface. This induces EC‐driven monocytic phagocytosis of the iRBC and excretion of parasitic toxins and hemozoin, the metabolically crystallized byproduct of hemoglobin digestion.^[^ ^19^, ^93^, ^94^ ^]^ Both secreted parasite proteins or toxins and host factors (cytokines, chemokines) cause significant direct effects on the brain endothelium leading to BBB disruption. This also leads to increased endothelial activation, apoptosis, oxidative stress, elevated secretion of cytokines and chemokines by monocytes and macrophages (immune cells), activation of platelets, and vascular hyperinflammation. More studies are needed to be done in the future to provide a conclusive evidence about the initiation/dominant step.

Malaria infection induces the production of reactive oxygen species (ROS) and NO. Once calcium‐activated endothelial nitric‐oxide synthase (eNOS) releases NO, it regulates vascular tone by causing vascular smooth muscle vasodilation and relaxation, thus maintaining endothelial stability. The mechanisms by which exogenous NO exerts its anti‐inflammatory effects have not been fully deciphered. It is, however, thought that NO prevents the activation of nuclear transcription factor kappa B (NF‐kB) through ROS scavenging, increased regulation and/or stabilization of its inhibitor I‐kB, as well as blocking the binding of the p50/p65 heterodimer.^[^ ^67^, ^95^, ^96^, ^97^, ^98^, ^99^ ^]^ Also, prostacyclin (or prostaglandin I2 (PGI2)), released from arachidonic acid by cyclooxygenase (COX‐1) in vascular EC, causes vascular smooth muscle relaxation and maintenance of vascular homeostasis.^[^ ^100^, ^101^ ^]^ These molecules contribute to the maintenance of an adequate environment for the endothelium. However, NO and PGI2 synthesis can be inactivated by increased cytokines and chemokines secretion under malaria infections, causing NO and PGI2 to exert vasoconstriction actions and contribute to endothelial dysfunction (ED).^[^ ^96^, ^102^ ^]^ Endothelin‐1,^[^ ^2^, ^60^ ^]^ angiotensin II,^[^ ^4^, ^5^, ^103^ ^]^ and thromboxane A2 ^[^ ^6^, ^7^ ^]^ activation during malaria infection can also upregulate eNOS expression, inducing increase expression of adhesion molecules and neutrophil adhesion to EC, provoking thrombosis. This will induce elevated cytokines/chemokines and ROS generation, vasoconstriction, and development of ED. Injury to the endothelium causes an imbalance between vasodilation and vasoconstriction, resulting in increased expression of leukocyte adhesion molecules, such as E‐ and P‐selectin, ICAM1, and vascular cell‐adhesion molecule 1 (VCAM1), increased platelet aggregation, leukocyte adhesion to the microvasculature, BBB damage, endothelial permeability and generation of NF‐kB‐mediated proinflammatory EC signature cytokines such as tumor necrosis factor‐alpha (TNF‐*α*), interferon gamma (IFN‐y), interleukin‐1 (IL‐1), IL‐6 (interleukin‐6) and chemokines such as the monocyte chemoattractant protein 1/chemokine ligand 2 (MCP‐1/CCL2) and CXCL8/IL‐8.^[^ ^8^ ^]^

### 3.2 The Role of Endothelial Cell‐to‐Cell Adhesions

EC junctions are composed of tight junctions, adherens junctions, and the platelet EC adhesion molecule‐1 (PECAM‐1, also known as CD31). These proteins maintain vessel wall integrity and regulate paracellular transport. Cerebral microvascular sequestration of iRBC increases adhesion of immune cells and platelets to the endothelium which can induce BBB breakdown. This results in infiltration of inflammatory cells (macrophages, B lymphocytes, T lymphocytes, and natural killer cells) into the brain tissue of malaria patients. The immune cells may further trigger an uncontrollable inflammatory cascade, causing further injury to the BBB.^[^ ^76^ ^]^ In fact, there is mounting evidence in CM and experimental cerebral malaria (ECM) that EC junction dysfunction significantly contributes to CM pathogenesis.^[^ ^24^, ^25^, ^26^, ^79^ ^]^ Positron emission tomography (PET) by Woodford J et al., computerized tomography (CT) scans by Potchen MJ et al., and magnetic resonance imaging (MRI), Seydel et al. observed morphological changes at EC‐EC junctions in post mortem brains,^[^ ^9^, ^10^, ^11^, ^13^, ^14^, ^15^ ^]^ which also exhibit increased sequestration of iRBC and leukocytes in the microvasculature, severe vasculopathy, increased endothelial activation and BBB dysfunction and disruption. In addition, there is persistent upregulation of adhesion molecules (ICAM‐1, VCAM‐1, E‐ and P‐selectins, and PECAM‐1), reduction in tight junction proteins (claudins, occludins, and ZO‐1), upregulation of inflammatory cytokines, reduced blood flow, vascular leakage, acute edema of both vasogenic and cytotoxic origin and microhemorrhages, leading to neurological impairment.^[^ ^16^, ^17^, ^60^ ^]^ Thus, the dysfunction of EC which is observed in CM likely is accompanied by PECAM‐1‐induced leukocyte trafficking which promotes a proinflammatory environment. During the erythrocytic schizogony iRBC undergo hemolysis, releasing plasmodial proteins such as parasite‐derived proteases dipeptidyl aminopeptidase 3 (Pf‐DPAP3) and Subtilisin‐like protease 1 (Pf‐Sub1), and human protease calpain‐1,^[^ ^19^, ^20^, ^104^ ^]^ as well as hemozoin and free hemoglobin from the host cell. Hemoglobin is oxidized by molecular oxygen to form methemoglobin (metHb), liberating the heme group which is pro‐inflammatory.^[^ ^21^, ^22^ ^]^ Elevated methemoglobin release and exposure to the ECs induces increase inflammatory and cellular adhesion molecules expression which promotes EC‐RBC adhesion. In addition to the cytotoxic free hemozoin and plasmodial proteins released by hemolysis (which induce cytokine release (TNF‐alpha and IL‐1)), the monocyte/macrophage system also generates ROS which contributes to cellular morphological changes and actin filament reorganization.^[^ ^23^ ^]^ These cellular changes result in increased paracellular permeability and enhanced infiltration of inflammatory cells (leukocytes and platelets) into the brain parenchyma.^[^ ^16^, ^24^, ^25^ ^]^

### 3.3 The Effect of Oxidative Stress on Endothelial Cells

**Figure 4:**
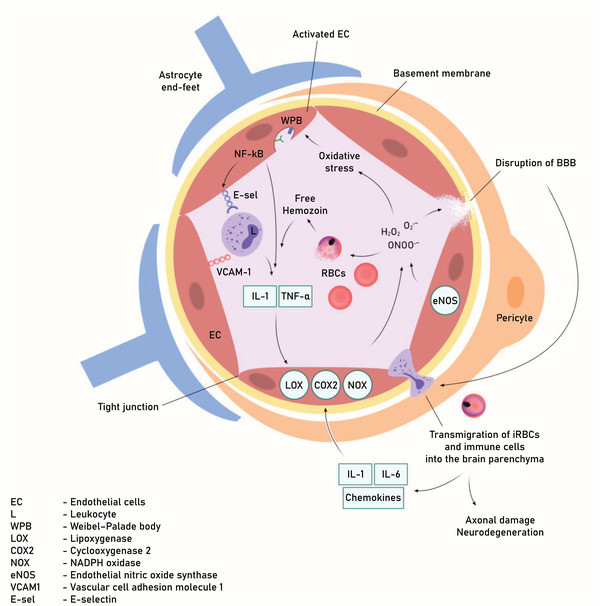
Pathophysiology of BBB leakage in CM. Increase systemic immune response against the malaria parasite causes increase circulating levels of pro‐inflammatory cytokines and chemokines. This inflammatory environment induces increase expression of adhesion molecules and secretion pro‐coagulation factors. These signals contribute to the recruitment of leukocytes. Increased cytoadherence of parasitized RBC to ECs via adhesion molecules (P‐ and E‐selectin, ICAM‐1, and VCAM‐1) compromises microvascular blood‐flow. Increase aggregation due to the secretion of pro‐coagulation factors (Tissue Factor, PAF, and vWF) promote intravascular coagulation. These mechanisms generate morphological alterations to activated ECs and contribute to BBB. dysfunction.

In CM, innate immune cells, despite being essential for antimalarial immunity, put EC under oxidative stress (**Figure** **4**). The host innate immune cells, monocytes, macrophages, and neutrophils, play a vital protective role in ROS response by upregulating antioxidant enzymes,^[^ ^105^ ^]^ increase phagocytosis (phagocytic oxidative burst), as well as oxidative stress induced by antimicrobial drugs, which are detrimental to *P. falciparum* parasites.^[^ ^28^, ^106^ ^]^ Increased production of ROS contributes to the clearance of the parasite. However, uncontrolled generation of ROS/reactive nitrogen species (RNS) by activated ECs can mediate elevated secretion of proinflammatory cytokines (TNF‐*α*, IL‐1, IL‐6) and chemokines (MCP‐1/CCL2, CXCL8/IL‐8) in the brain parenchyma.^[^ ^29^ ^]^ This may result in excessive inflammation or hyperinflammation, damage to host neuronal cells, and probably contributes to severe neurodegeneration.^[^ ^30^, ^31^ ^]^ These multiple sources of oxidative stress (from host innate immune cells, free hemozoin, and antimalarial drugs) contribute to ECs activation by inducing the cells into a proinflammatory and procoagulant state which, in concert with increased leukocyte interactions, culminates in the release of Weibel‐Palade bodies.^[^ ^32^, ^33^, ^34^, ^35^ ^]^ These storage organelles reside in EC and can be rapidly released via exocytosis, which is an important early step in vascular inflammation and thrombosis.^[^ ^33^, ^36^ ^]^ These cellular interactions between ECs and immune cells (leukocytes, macrophages neutrophils) eventually cause ED and BBB rupture, followed by increased endothelial permeability. All of these factors play an important role in tissue edema formation and leukocyte extravasation into the brain parenchyma during CM.^[^ ^37^, ^38^, ^39^ ^]^ Several studies have discussed the major pathways of oxidative stress in CM pathogenesis including hemodynamic stress, ED, blood vessel wall remodeling, and apoptotic cell death.^[^ ^40^, ^41^ ^]^ These major pathways play a critical role in contributing to the formation of ROS, such as superoxide (O2^•−^), hydrogen peroxide (H_2_O_2_), and peroxynitrite (ONOO^•−^).^[^ ^35^ ^]^ The main enzymatic sources of ROS in the cerebral vasculature include cyclooxygenase‐2 (COX‐2), lipoxygenase, and NADPH oxidase.^[^ ^40^, ^42^ ^]^ NADPH oxidase is used by brain EC and macrophages to produce O2^•−^ and H_2_O_2_ in response to parasite infection, cytokines, chemokines, and hemostatic dysfunction.^[^ ^43^ ^]^ The COX‐2 pathway is a major source of O2^•−^ generation in response to IL‐1 and TNF‐*α*.^[^ ^42^ ^]^ Other studies have demonstrated that cyclooxygenase and lipoxygenase pathways may be important sources of free radicals in malaria.^[^ ^44^, ^45^ ^]^ Extravasation of hemozoin and immune cells leads to the production of high levels of free radicals such as ROS, and cytokines/chemokines which inflicts damage to the membrane of the RBC, reducing their deformability and increasing their rigidity, cumulating in anemia.^[^ ^46^, ^47^ ^]^ Also, increased oxidative stress induces lipid peroxidation. This provokes functional and structural changes to the plasma membrane that lead to hemolysis, ECs activation, and dysfunctions. Higher levels of the lipid peroxidation marker, malondialdehyde (MDA), and decreased levels of antioxidants are all indications that oxidative stress plays a crucial role in controlling *P. falciparum* parasite infection.^[^ ^48^ ^]^

Studies have demonstrated that ROS and reactive nitrogen intermediates (RNI) produced during phagocytosis (respiratory burst) are cytotoxic for parasites by crossing and causing damage to iRBC membranes and ECs. This major source of ROS in the cerebral vasculature is comprised of three main isoforms: neuronal NOS (nNOS), inducible NOS (iNOS), and endothelial NOS (eNOS).^[^ ^67^ ^]^ eNOS is more abundant in vascular EC.^[^ ^49^, ^50^, ^65^ ^]^ ROS are formed through endothelial nitric oxide synthase (eNOS) uncoupling and as a result, eNOS commences to produce O2^•−^.^[^ ^51^, ^52^ ^]^ The production of adequate levels of NO in the vascular endothelium is critical.^[^ ^98^ ^]^ Increased eNOS produced by NO functions as a blood flow regulator by inducing blood vessel vasodilatation, preventing platelet aggregation, and inhibiting adhesion of leukocytes and monocytes to the endothelium, thus preventing ischemia and improving microcirculation.^[^ ^50^, ^53^, ^54^, ^65^, ^98^ ^]^

Reduced vascular NO availability would increase ROS generation and induce cellular responses in different circumstances (disease state, metabolic alterations, etc.).^[^ ^55^ ^]^ NO produced during cytokine‐stimulated blood vessel inflammation may diffuse through the BBB, causing synaptic dysfunction and contributing to neurodegeneration.^[^ ^56^ ^]^ The incapacity to overcome the generation of ROS or the inability to generate an adequate antioxidant response could induce vascular ED in CM, the outcome being alterations to endothelial signal transduction and redox‐regulated transcription factors.^[^ ^37^ ^]^ The transcription factor nuclear factor kappa‐light‐chain‐enhancer of activated B cells (NF‐*κ*B) induces and regulates the expression of various genes involved in cell proliferation, adhesion, anti‐apoptosis, and apoptosis.^[^ ^57^, ^107^ ^]^ As a response to vascular inflammation and oxidative stress, activation of NF‐*κ*B via protein kinase C (PKC) and mitogen‐activated protein kinase (MAPK)‐dependent pathways upregulates the secretion of proinflammatory cytokines TNF‐*α*, IL‐6, IL‐1, and chemokines (IL‐8). NF‐kB also regulate upon activation, normal T cell expressed and presumably secreted (RANTES)) and the proinflammatory enzymes COX‐2 and iNOS.^[^ ^42^, ^59^, ^60^, ^108^ ^]^ Studies have demonstrated that increased expression of adhesion molecules on the endothelium correlates to higher NF‐*κ*B activation. Malaria infection induces increased activation of NF‐*κ*B, which upregulates EC adhesion molecules that mediate leukocyte rolling (E‐selectin) and adherence (ICAM‐1, VCAM‐1).^[^ ^57^, ^62^, ^107^ ^]^ Elevated TNF*α* secretion triggers the upregulation of endothelial adhesion molecules ICAM‐1, VCAM‐1, or E‐selectin expression. This potentiates the effects of increased leukocyte recruitment and adhesion, thus allowing malaria antigens and immune cell infiltration into the brain parenchyma. Following transmigration of iRBC and immune cells into the brain parenchyma as a result of BBB disruption in murine and humans during CM, cytokines (IL‐1, IL‐6) and chemokines (MCP‐1/CCL‐2, CXCL8/IL‐8) secretion from different immunocompetent cells, such as neurons, microglia, astrocytes, and cerebrovascular ECs is increased. Chronic activation of these immunocompetent cells by cytokines/chemokines further potentiates ROS production via NADPH oxidase, resulting in an inflammatory cytokine storm.^[^ ^63^, ^64^ ^]^ The result is increased activation and insult/injury to immunocompetent cells, resulting in axonal damage, neurodegeneration, and neurocognition impairments.^[^ ^38^, ^60^, ^66^, ^68^, ^109^, ^110^ ^]^

### 3.4 Interaction between Endothelium and Immune Cells

Increased expression of cellular adhesion molecules (CAMs) caused by excessive activation of the endothelium further enhances the adhesion of other inflammatory cell types, such as neutrophils, leukocytes, and platelets to the EC surface, causing coagulation.^[^ ^69^, ^70^, ^71^, ^72^ ^]^ Inflammatory stimuli (e.g., TNF, IL‐1*β*, and IFN‐*γ*) released by these immune cells serve as critical mediators of BBB disruption in CM. Proinflammatory signaling induces cell adhesion molecules and subsequent transmigration of activated neutrophils, lymphocytes, or monocytes into brain parenchyma,^[^ ^73^, ^75^, ^111^ ^]^ a commonly observed characteristic in CM postmortem brains. These immune cells, together with sequestered platelets in the brain parenchyma, activate ECs and induce uncontrolled secretion of inflammatory cytokines (TNF‐*α*, IL‐1 Endothelin‐1 (ET‐1), IFN‐y) and chemokines (MCP‐1/CCL‐2, angiopoietin‐1/2 (Ang‐1/2)), vWF and tissue factor (TF) during CM.^[^ ^60^, ^61^, ^76^, ^78^, ^112^ ^]^ Both Ang‐1/2 are produced in ECs and pre‐stored in the Weibel–Palade bodies (WPB) together with vWF. Ang‐1 (control vascular quiescence and stability) and Ang‐2 (promote vascular permeability and proinflammatory microvascular dysfunction) are both ligands of the Tie‐2 receptor, which is expressed on ECs.^[^ ^79^ ^]^ Upon activation of ECs during CM, exocytosis of the WPB is induced and their content is released into the blood. vWF and Ang‐1/2 release may have extended effects in further activation of ECs by activation of a pro‐inflammatory amplification loop to effect ECs dysfunctions.^[^ ^80^ ^]^ In addition, excessive secretion of IL‐1 causes the EC to release chemokines, such as MCP1/CCL2 and cytokine ET‐1, which induces further recruitment of leukocytes to the brain.^[^ ^81^, ^82^, ^83^ ^]^ Increased expression of ET‐1 further activates the endothelium and upregulates the expression of CAMs,^[^ ^84^ ^]^ demonstrating that ET‐1 increases recruitment of iRBC, leukocytes, and platelets to the cerebral vasculature,^[^ ^85^, ^86^ ^]^ causing microvascular obstruction, thrombosis and vasoconstriction. These cellular driven proinflammatory mechanisms exacerbate BBB injury and permeability, increasing the likelihood of hemorrhages and petechiae which is characteristic in the brains of CM individuals.

An increased immune response is one of the major contributors to CM vasculopathy. Circulating levels of TNF‐*α* are significantly higher in human CM and ECM mice, compared with those that do not develop the neurological syndrome.^[^ ^87^ ^]^ Elevated TNF‐*α* and IFN‐y further induce EC activation and are responsible for the consequent upregulation of the expression of several CAMs, including ICAM‐1 (CD54), CD36, P‐selectin (CD62P), and VCAM‐1 (CD106).^[^ ^22^, ^87^, ^88^ ^]^

### 3.5 Interaction between Vascular Endothelium and Platelets

**Figure 5:**
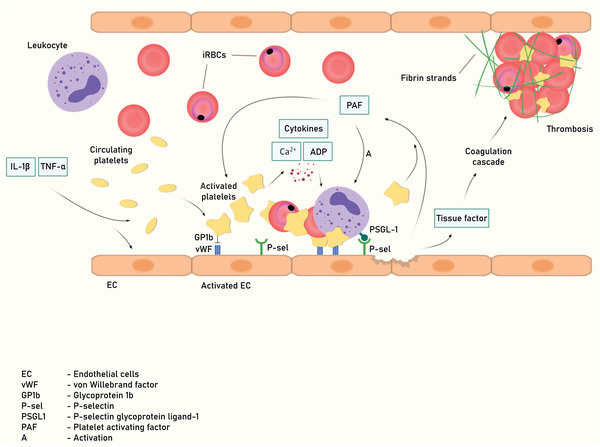
Interaction between brain vascular endothelium and platelets during CM. During cerebral malaria, increase adhesion of iRBC and leukocytes (mainly mononuclear cells) to brain vasculature result in capillary congestion. This provokes an increase secretion of proinflammatory cytokines, increase of cellular adhesion molecules expression, release of hemozoin from ruptured iRBC, decrease of NO, ECs dysfunction, and BBB. Transmigration of iRBC and immune cells into the brain parenchyma activates microglial cells and astrocytes. Excessive secretion of inflammatory cytokines and chemokines and decrease of NO, COX and LOX further exacerbate ECs activation and BBB dysfunction.

Another important aspect of inflammation in CM patients is the induction of abnormal intravascular coagulation and of thrombi (**Figure** **5**).^[^ ^89^ ^]^ ECs activated by high IL‐1*β* and TNF‐*α* secretions during CM can trigger coagulation by displaying vWF, P‐selectin, and fibrinogen onto which platelets bind. Platelets contain 3 distinct types of granules: *α*granules, dense or *δ*‐granules, and lysosomes.^[^ ^113^, ^114^, ^115^ ^]^ Also, upon activation in CM state, platelet *α*‐granules secrete various proteins, chemokines, cytokines, and growth factors, *δ*‐granules secrete adenosine diphosphate (ADP), serotonin, polyphosphates, glutamate, histamine, and calcium, and lysosomes secrete glycohydrolase enzymes.^[^ ^32^, ^59^, ^93^, ^94^, ^96^, ^116^ ^]^ These secreted molecules further enhance platelet aggregation with nearby leukocytes and iRBC, sequestering and binding them to vascular EC and damaging the microvessel at the site of injury which is indicated by the elevated expression of adhesion molecules (VCAM‐1 and ICAM‐1), P‐selectin and L‐ and E‐selectins.^[^ ^97^ ^]^ The sequestered iRBC further prevents normal blood circulation, inducing vascular EC to activate the extrinsic and intrinsic pathways of blood coagulation by secreting elevated levels of tissue factor (TF), which promotes the initiation of the coagulation cascade. This results in the conversion of fibrinogen to fibrin which cumulates in the development of a thrombosis.^[^ ^70^, ^98^ ^]^ Another important mediator, the vWF, is released by Weibel‐Palade bodies.^[^ ^33^, ^36^ ^]^ Elevated vWF secretion causes increased recruitment of platelets by activated EC, thus leading to widespread coagulation and inflammation.^[^ ^32^, ^69^, ^99^ ^]^ The platelet‐activating factor (PAF) is also an important inflammatory mediator associated with CM. PAF recruits and activates leukocytes, increases cytokine and chemokine secretion, and modulates vascular permeability.^[^ ^117^, ^118^ ^]^

### 3.6 Endothelial Disruption and Subsequent Effects on Astrocytes, Neurons, and Microglia

Microglia are CNS‐resident immune cells with multifaceted functions during normal physiological and CM pathological conditions. Astrocytes, neurons, and microglia respond to *P. falciparum* or CM (from *P. falciparum* infection) by exhibiting reactive phenotypes and immune responses.^[^ ^119^ ^]^ Upon disruption of the BBB, there is extravasation of iRBC, soluble and insoluble parasite products, leukocytes, cytokines, and chemokines into the brain parenchyma.^[^ ^120^, ^121^ ^]^ These molecules and proteins have been observed to activate microglia and astrocytes in murine and human CM,^[^ ^120^, ^122^ ^]^ causing further secretion or release of active mediators (cytokines, chemokines) by recruited immune cells (leukocytes, neutrophils) and platelets, resulting in hyperinflammation. Parasite and host proteins cause activated astrocytes and microglia cells in ECM ^[^ ^123^, ^124^ ^]^ to release cytokines (IL‐1, IL‐6, TNF‐*α*), chemokines (endothelin‐1, CCL2), and ROS. BBB dysfunction amplifies the development of neuroinflammation, rather than being a pathological result of glial activation and neuroinflammation. Therefore, chronic and sustained neuroinflammation caused by prolonged glial ^[^ ^125^ ^]^ and astrocyte ^[^ ^126^ ^]^ activation has been reported to culminate in neuronal death, exhibiting a correlation with brain defects associated with CM. Astrocytes play a vital role in maintaining BBB properties and survival of neurons.^[^ ^120^ ^]^ In CM, reactive astrocytes, pericytes, and microglia (during gliosis) also secrete inflammatory factors such as vascular endothelial growth factor A (VEGF‐A), MMPs, transforming growth factor‐beta (TGF‐*β*), and MCP‐1, aggravating BBB disruption and recruiting more immune cells.^[^ ^127^, ^128^ ^]^ This could ultimately lead to the neuronal damage and cognitive impairments observed in CM.^[^ ^73^, ^75^ ^]^ Chronic inflammation may result in functional impairments of astrocytes and consequently, be detrimental to normal neuronal and synaptic activity.^[^ ^73^ ^]^

## 4 Pathogenesis, Diagnosis, and Treatment of Cerebral Malaria

### 4.1 Pathogenesis of Cerebral Malaria

CM is the most severe and fatal neurological complication of infection with *P. falciparum*,^[^ ^129^, ^130^ ^]^ and can be defined as the presence of peripheral *P. falciparum* parasitemia and coma with no other apparent causes of altered consciousness.^[^ ^87^, ^131^, ^132^ ^]^

African children, Asian adults, pregnant women, and immunodeficient individuals are the most affected, with most cases being reported for children under 5 living in the WHO African Region.^[^ ^79^, ^133^, ^134^, ^135^ ^]^ CM in children and adults is clinically characterized by impaired consciousness, severe anemia, hypoglycemia, fever, and neurocognitive sequelae as well as diffuse encephalopathy with a deep unarousable coma (Blantyre coma score of ≤ 2), high parasitemia, hypoxia, meningitis and a postictal state of the patient.^[^ ^136^, ^137^, ^138^, ^139^, ^140^, ^141^ ^]^ The clinical course of CM appears to be wide‐ranging, with adolescents accounting for ≈70–75% of malaria‐related deaths in Africa.^[^ ^87^, ^135^, ^142^, ^143^, ^144^, ^145^ ^]^ The mortality rate significantly decreases as a result of proper treatment, but is still around 20%.^[^ ^142^, ^146^, ^147^, ^148^ ^]^ ≈25% of survivors develop long‐term neurological and cognitive deficits such as behavioral abnormalities, learning and speech difficulties, impaired motor functions, and a higher rate of seizures.^[^ ^87^, ^141^, ^143^, ^147^, ^149^, ^150^, ^151^, ^152^, ^153^ ^]^

The etiology of CM is not completely known. In adult CM cerebral sequestration and microvascular obstruction is suggested to lead to coma and earlier death before treatments.^[^ ^132^ ^]^ Pediatric CM can be classified into two types, CM1 and CM2.^[^ ^79^, ^154^, ^155^ ^]^ Clinical and pathological diagnosis and analysis have shown that CM1 exhibits increased iRBC sequestration in the brain microvasculature endothelium, while CM2 exhibits increased parasite growth and iRBC sequestration, increased coagulation and congestion, occlusion of capillaries and venules, inflammation and brain endothelial disruption (due to the disruption of tight junctions) as well as extensive microhemorrhages and petechiae in the brain parenchyma.^[^ ^25^, ^142^, ^156^, ^157^ ^]^ This results in increase fluid leakage, vasogenic edema, and herniation of the brain. Pediatric CM is associated with a higher rate of seizures and post‐CM cognitive deficits.^[^ ^19^, ^142^, ^143^, ^158^, ^159^ ^]^ Brain swelling causes an increase in intracranial pressure, contributing to the pathogenesis of CM in children. Clinical data have shown brain images consistent with a compromised brainstem due to increased intracranial pressure.^[^ ^142^, ^160^ ^]^ In one study, CT scans showed increased brain volumes in 6 of 14 children.^[^ ^161^ ^]^ The pathogenesis of CM in humans has some differences to that in animal models, leading to debates and discussions about the utility of animal models and their applicability to human CM pathophysiology.^[^ ^162^ ^]^ Several differences between human CM and ECM caused by the murine malaria parasite *Plasmodium berghei* (*P. berghei*) include less sequestration of iRBC and the accumulation of immune cells (leukocytes, monocytes, macrophages, and T cells) in the brains of mice with ECM.^[^ ^163^, ^164^ ^]^

### 4.2 Diagnosis of Cerebral Malaria

It is noted clinically that early symptoms of CM can progress rapidly to increased intracranial pressure, hemiparesis, ataxia, and coma. If immediate medical treatment is not provided, the risk of treatment failure and mortality increases, especially in children. CM diagnosis (**Table** **1**) is beset with several difficulties, especially in a Tropical and Sub‐Tropical environment with a broad spectrum of parasitic diseases. In addition to CM misdiagnosis, CM diagnosis is hampered by the difficulty of studying the human brain in vivo. In malaria‐endemic regions the challenge is further worsened by lack of reliable infrastructure and imaging. Solving malaria infections demands a proper diagnosis of asymptomatic and symptomatic malaria infections.

**Table 1:** Diagnosis of Cerebral Malaria

Diagnostic method	References
Thin and thick blood smears	[3]
Rapid diagnostic test (RDT)	[3]
Polymerase chain reaction (PCR)	[3]
Malaria retinopathy	[174, 175, 176]
Lumbar puncture	[177]
Fundoscopy (direct and indirect retinoscopy)	[142, 174]
Electroencephalogram (EEG)	[314]
Cerebral fluid sample (CFS)	[315]
Cerebral imaging (MRI)	[316]

The most common form of diagnosis requires the demonstration of asexual developmental forms of *P. falciparum* in peripheral blood using Giemsa‐stained thick and thin blood smears (for severe malaria and CM, respectively), which may be followed by imaging or post‐mortem histopathology to determine blood obstruction in the brain.^[^ ^79^, ^142^, ^165^ ^]^ Other tests include the immunochromatographic test for *P. falciparum*, histidine‐rich protein 2 and lactate dehydrogenase,^[^ ^166^ ^]^ dipstick, cassette or hybrids which detect evidence of malaria parasites (antigens),^[^ ^167^ ^]^ and a polymerase chain reaction (PCR) test for parasite messenger RNA or DNA.^[^ ^168^, ^169^, ^170^ ^]^ These tests are more sensitive than microscopy but are also more expensive. Although severe malaria is clearly linked to higher parasitemia, the amount of *P. falciparum* parasite sequestration and vascular congestion correlates with CM disease severity.^[^ ^171^, ^172^, ^173^ ^]^ However, parasitemia and parasite sequestration are not the only prerequisites of CM.

Currently, an efficient and safe way to evaluate CM blood obstruction is through assessment for malarial retinopathy by retinal observation.^[^ ^174^, ^175^, ^176^ ^]^ The presence of malarial retinopathy is an important clinical feature that distinguishes CM subjects from those with alternative pathologies, such as meningitis. To further confirm CM diagnosis in most patients, lumbar puncture is highly recommended in other to exclude other bacterial infections.^[^ ^177^ ^]^ Fundoscopy through direct or indirect retinoscopy is used in malaria endemic areas to help clinicians distinguish CM from other causes of encephalopathy.^[^ ^142^, ^174^ ^]^ Use of cerebral imaging of patients with CM might be necessary for appropriate clinical diagnosis.^[^ ^82^, ^143^, ^178^ ^]^ The pathogenesis of retinopathy includes sequestration of iRBC cells in retinal and cerebral microvasculature, causing vessel obstruction and reduced blood flow.^[^ ^179^ ^]^ This results in retinal hemorrhages, hypoxia, and retinal whitening.^[^ ^174^, ^180^, ^181^ ^]^ Brain autopsies of children with retinopathy‐positive CM have demonstrated increased levels of sequestration of iRBC in cerebral vasculature, while retinopathy‐negative CM patients have less iRBC sequestration in the cerebral vasculature.^[^ ^142^, ^148^ ^]^ In addition, MRI studies have shown that brain swelling is correlated with BBB breakdown and death.^[^ ^9^, ^182^, ^183^ ^]^ The sequestration and accumulation of iRBC in the cerebral capillaries cause mechanical obstruction of the vessels leading to a reduction in blood flow, hypoxia, coma, and death due to increased intracranial pressure, acute respiratory arrest, and fatal brainstem herniation.^[^ ^132^, ^184^ ^]^ Postmortem analysis demonstrates that sequestered parasites, immune cells, and cytokine/chemokine secretion causes injury to the brainstem and small lesions, which may be the cause of death.^[^ ^87^ ^]^ Most studies and observations regarding human CM have been done on human postmortem brains, which are not readily available due to ethical constraints. Therefore, animal models, despite their imperfections, are often used for longitudinal studies of CM disease pathogenesis.^[^ ^129^ ^]^ In fact, most studies carried out in non‐human primate models have reported pathological features similar to those in humans, including cerebral microvasculature sequestration of iRBC, vascular damage and BBB disruption, and persistent cognitive impairment despite successful antimalarial therapy ^[^ ^60^, ^84^, ^131^, ^185^ ^]^ (**Table** **2**).

**Table 2:** Models to investigate Cerebral Malaria

Models to investigate CM	References
2D PAMPA (parallel artificial membrane permeability assays)	[317]
2D Transwell models	[282]
3D organoids	[287, 318, 319]
3D‐perfusable BBB organ‐on‐a‐chip models	[320, 321, 322]
3D‐spheroid models	[93, 323]
3D‐perfusable micro‐ and macro‐vascular models	[284, 313]
3D bioprinted structures	[323, 324]
In vivo‐models (murine)	[74, 163, 310]

CM is fatal when left untreated.^[^ ^186^ ^]^ Postmortem analyses of fatal CM brains have revealed blood vessel occlusions with iRBC. Electron microscopy analyses have further shown that most ECs were swollen, with some containing the malaria pigment hemozoin,^[^ ^121^ ^]^ which strongly suggests extravasation and internalization of iRBC into the brain parenchyma.^[^ ^79^, ^93^, ^187^ ^]^

The breakdown of the BBB is an important feature of CM. The BBB is disrupted when BMVECs are activated by sequestered iRBC, platelets, and macrophages,^[^ ^16^ ^]^ leading to severe neurological complications amongst which are intracerebral hemorrhage, electrolyte imbalance, and seizures, increase in intracranial pressure, edema and axonal damage. However, the precise underlying mechanisms leading to the disruption of BBB integrity during CM remained unclear until recently.^[^ ^188^ ^]^ A study published in 2021 by Adams et al. revealed that EC of the brain can take up iRBC in an ICAM‐1‐dependent manner, which ultimately results in swelling of the EC and breakdown of the BBB.^[^ ^93^ ^]^

Several studies have demonstrated widespread endothelial activation during CM. However, how ED results in CM is poorly understood. Although the underlying mechanisms are multicellular and multifactorial, a combination of microvascular and immune system dysfunctions are common.^[^ ^75^, ^88^ ^]^

### 4.3 Therapeutic Approaches and Treatments against Cerebral Malaria

Severe malaria is a complex multi‐system disease and clinical symptoms associated with CM are not easy to identify. The clinical manifestations are similar in adults and children, but it can be difficult to differentiate CM from encephalitis, meningitis, and other febrile convulsions. However, the most common and feared complications of CM are severe impairment of consciousness (deep coma), general malaise, headache, fits, vomiting, and diarrhea.^[^ ^189^ ^]^ Initiation of treatment as a prophylactic measure or after CM diagnosis is crucial.

Currently, prophylactic antimalarials (pyrimethamine, proguanil, and primaquine) are administered to immunocompromised individuals traveling from non‐malaria countries to malaria‐endemic countries.^[^ ^190^ ^]^ Antimalarial drugs are also the only therapeutic option for patients who present with the complications of CM. Furthermore, the lack of understanding of the pathogenesis of CM hinders identification of new drug targets for therapeutic intervention. Therefore, there is an urgent need to develop adjunctive therapies that can be administered with currently available antimalarials that can both halt the progression of CM and prevent or reverse BBB dysfunction. Fortunately, the WHO has established evidence‐based guidelines for the treatment of CM. It includes early diagnosis, prompt and effective treatment of malaria, rational use of antimalarials agents, combination therapy, and appropriate weight‐based dosing.^[^ ^3^, ^10^, ^191^, ^192^, ^193^ ^]^

Antimalarial compounds such as artemisinin (ART, also known as Qinghaosu) and/or artemisinin‐based combination therapies (ACTs) have shown evidence of improving the patients’ health (symptoms, disease duration, and/or survival) as well as preventing malaria parasite resistance.^[^ ^194^, ^195^ ^]^ In addition, administration of antibodies from convalescent plasma,^[^ ^196^ ^]^ anti‐inflammatory agents (dexamethasone),^[^ ^197^, ^198^ ^]^ immunomodulatory therapies,^[^ ^199^, ^200^ ^]^ and anticoagulants such as heparin ^[^ ^201^ ^]^ have also demonstrated to reduce the risk of mortality and neurocognitive sequelae in patients with CM. In CM, artemisinins induce immunosuppression by downregulating proinflammatory cytokines and chemokines of both the innate and acquired immune systems.^[^ ^202^ ^]^ Artemisinin and its synthetic derivatives (dihydroartemisinin, artesunate, artemether, and arteether) contain an intramolecular endoperoxide bridge situated in the sesquiterpene lactone backbone structure.^[^ ^203^ ^]^ This endoperoxide bridge is responsible for their antimalarial activity and must be activated to generate free radical species.^[^ ^203^, ^204^, ^205^, ^206^ ^]^ However, treatment with high dosage or concentration of artemisinin‐derivatives alone does not prevent death or neurological disability in most CM subjects.^[^ ^207^, ^208^ ^]^ Therefore, drugs such as chloroquine, quinine, and mefloquine, which are typical fast acting schizonticidal drugs, can be used to prevent death and/or mitigate neurological impairments in CM. Despite their schizonticidal activity, the use of pyrimethamine, sulphonamides, and sulphone in CM is limited by their slow metabolic actions.^[^ ^209^ ^]^ Quinoline derivatives are less effective than artesunate and artemisinin derivatives (artemether/lumefantrine, artesunate/amodiaquine, artesunate/mefloquine or dihydroartemisinin/piperaquine) for the treatment of severe malaria due to parasite resistance.^[^ ^210^, ^211^, ^212^ ^]^ WHO has therefore recommended the combination of quinoline derivatives (chloroquine, mefloquine, quinine, and quinidine) and artemisinin‐based combination therapies (ACT).^[^ ^212^, ^213^ ^]^ Artemisinin derivatives act quickly against the intraerythrocytic asexual blood‐stage malaria parasites but have very short in vivo half‐lives of ≈1–3 h (typically ≈1 h in humans).^[^ ^214^, ^215^, ^216^ ^]^ In order for artemisinin or its derivatives to be effective in CM, they must be co‐administered with other antimalarial drugs with longer half‐lives.^[^ ^212^ ^]^ Artemisinin derivatives prevent CM neurocognitive complications by mitigating or preventing ED, which is a key event in CM pathogenesis.^[^ ^217^, ^218^, ^219^ ^]^ Combined administration of artesunate and tetramethylpyrazine has an adjuvant therapeutic effect on the symptoms of ECM by modulating NO bioavailability in the brain.^[^ ^220^, ^221^ ^]^ Artesunate and tetramethylpyrazine administration increases NO bioavailability, reduces EC injury, improves cerebral blood flow, and inhibits platelet and lymphocyte activation, thereby preventing vascular leakage and metabolic complications.^[^ ^222^, ^223^ ^]^ Currently, artesunate is widely accepted as the standard treatment for CM.^[^ ^191^, ^192^, ^193^ ^]^

Furthermore, adjunctive therapy in CM is aimed at targeting *P. falciparum* directly as well as modulating the host response to infection. This approach might reduce malaria‐associated morbidity and mortality by improving the effects and efficiency of current antimalarial compounds, thereby reducing complications associated with CM.^[^ ^224^ ^]^ For example, in other to reduce brain swelling and inflammation, corticosteroids (dexamethasone),^[^ ^225^, ^226^, ^227^ ^]^ intravenous immunoglobulin,^[^ ^228^ ^]^ curdlan sulfate (CS), a sulfated 1 → 3‐*β*d glucan,^[^ ^229^, ^230^ ^]^ anti‑TNF therapy ^[^ ^231^, ^232^, ^233^ ^]^ and peroxisome proliferator‐activated receptor‐*γ* (PPAR‐*γ*) agonists ^[^ ^234^, ^235^, ^236^, ^237^, ^238^ ^]^ have been proposed and used in both CM and ECM as adjunctive therapies with contrasting results.^[^ ^224^ ^]^ Administration of dexamethasone did not reduce mortality in two clinical trials,^[^ ^225^ ^]^ while intravenous administration of immunoglobulin increased mortality and worsened neurological sequelae.^[^ ^200^, ^228^ ^]^ On the contrary, curdlan sulfate (CS), a sulfated glycoconjugate compound, is administered through intravenous infusion in CM and has a short plasma half‐life (2 to 3 h).^[^ ^239^ ^]^ Clinical trial data have shown that CS exhibits some direct and non‐specific effects through cytoadhesion and rosetting inhibition of infected and uninfected RBC on EC, thereby reducing CM severity, although no differences in mortality were observed.^[^ ^230^ ^]^ The free radical scavenger edaravone inhibits cerebral aneurysm (CA) formation in animal studies by preventing astrocyte and glial activation and mitigating programmed death of neuronal cells.^[^ ^24^, ^240^, ^241^ ^]^ Since 2001 edaravone is administered in Japan and most countries in Asia, but only recently in USA and Canada (2017 and 2018). Edaravone, a free radical scavenger, is used for the treatment of neurodegenerative diseases (such as ALS, Parkinson's disease, AD, and cerebral infarction).^[^ ^241^ ^]^ Edaravone offers neuroprotection under oxidative stress by inhibiting or preventing excessive ROS production and mitigating astrocyte and glial activation.^[^ ^240^ ^]^ Although edavarone is not currently administered in infectious disease, there is speculation that it may help prevent severe malaria disease through targeting of ROS, and therefore offer a promising new avenue for future bench to clinic studies and CM neuronal damage prevention.^[^ ^241^, ^242^, ^243^ ^]^ Targeting endothelial activation and preventing microvascular permeability, vascular leakage and BBB injury during CM could also be a potential target for adjunctive therapy to decrease CMinduced inflammation and neurocognitive dysfunction.^[^ ^36^ ^]^ The angiopoietin‐Tie2 (Ang1/Tie2) axis regulates EC function and vascular integrity.^[^ ^76^, ^244^, ^245^ ^]^ Increased Ang‐1 promotes EC quiescence and survival via its interaction with endothelial receptor Tie‐2. During CM, increased Ang‐2 levels inhibit Tie‐2, induce EC activation, and may result in endothelial dysregulation and microvascular leak.^[^ ^244^, ^246^, ^247^, ^248^ ^]^ Elevated Ang‐2 and low Ang‐1 levels are common in severe and CM. These imbalances in Ang‐1, Ang‐2, and soluble Tie2 concentrations have been demonstrated to correlate to disease severity and death in CM in both murine models and human infections.^[^ ^76^ ^]^ Indeed, Higgins et al. demonstrated that adjunctive administration of recombinant Ang‐1 to Ang‐1‐deficient mice prevented BBB breakdown during infection and improved survival in CM.^[^ ^249^ ^]^ Other therapeutic strategies to strengthen vascular barrier integrity are currently in use.^[^ ^250^ ^]^ Administration of sphingosine‐1‐phosphate (S1P), a sphingolipid released from platelets, or its drug analog, prevented vascular injury and dysfunction in various infectious diseases. For example, both FTY720 (a potent S1P receptor agonist) alone or in combination with artesunate improved clinical outcomes in human patients and in a murine model of CM.^[^ ^251^ ^]^

Statins are also used for adjunctive therapy. They exhibit anti‐inflammatory actions (by reducing the pro‐inflammatory effects of macrophages and neutrophils) and attenuate neuronal damage through prevention of EC activation and cerebral ischemia.^[^ ^252^ ^]^ Statins function through reduction of iNOS induction and expression and increase of eNOS release. Statins reduce the expression of endothelial adhesion molecules (ICAM‐1, VCAM‐1, and E‐selectin) ^[^ ^253^ ^]^ and may inhibit *P. falciparum* cytoadherence and endothelial damage.^[^ ^254^ ^]^ For example, atorvastatin prevents expression of C‐X‐C motif chemokine ligand 10 (CXCL10), also known as IFN‐*γ*‐induced protein 10 (IP‐10), and therefore reduces CM mortality in adult CM patients.^[^ ^255^, ^256^ ^]^ Upregulation of CXCL10 is induced by inflammatory molecules (IFN‐*γ* and TNF‐*α*) which leads to the chemoattraction of monocytes and the adhesion of T cells to EC, eventually causing EC dysfunction.^[^ ^255^, ^256^ ^]^ In ECM, some protection could be achieved by treating CXCL10deficient mice with atorvastatin. The survival rates of the atorvastatin treated mice improved and brain tissue analysis showed increased transcription of Ang‐1 and reduced levels of Ang‐2.^[^ ^257^, ^258^ ^]^

Another way to treat or even prevent CM could be the direct targeting of PfEMP1 presented on the surface of iRBC. PfEMP1 proteins are classified into three major groups, A, B, and C.^[^ ^259^, ^260^ ^]^ The ICAM‐1‐binding group A PfEMP1 proteins tend to bind the EPCR which is associated with adhesion of iRBC to vascular EC, causing ED and CM in children.^[^ ^261^, ^262^ ^]^ Targeting intracerebral sequestration of iRBC with antibodies against the DBL*β* motif of PfEMP1 and EPCR ^[^ ^263^, ^264^, ^265^ ^]^ in combination with rapamycin seems to be effective in reducing the expression of EC receptors such as ICAM‐1, VCAM‐1, E‐selectin, EPCR and CD36.^[^ ^21^, ^23^, ^266^ ^]^ The result is the prevention of microvasculature occlusion, BBB dysfunction, and EC activation. Also, treatment of mice infected with the ANKA strain of *P. berghei* with neuregulin‐1 (endothelial barrier stabilizer) protected the animals from developing ECM by reducing the levels of proinflammatory cytokines TNF‐*α*, IL‐6 and IL‐1 while inducing endogenous Ang‐1 (antiinflammatory pathway) and CXCL10, which then decreased the accumulation of leukocytes in the brain and inhibited vascular permeability by stabilizing EC tight junctions.^[^ ^256^, ^267^ ^]^

The dysregulation of coagulation occurs frequently in patients suffering from CM. This thrombotic effect may be caused by reduced expression of the iRBC‐binding receptor EPCR and the anticoagulant/thrombin receptor thrombomodulin.^[^ ^268^ ^]^ Once the coagulation cascade is triggered by iRBC, TF is released by activated EC, eventually leading to thrombosis.^[^ ^70^, ^269^ ^]^ Therefore, therapies aimed at preventing platelet dysfunction and ED should also be considered.^[^ ^221^, ^268^ ^]^ Targeting the coagulation pathways with compounds such as heparin seems to be effective against venous thromboembolism because it prevents rosette formation and cytoadherence to EC.^[^ ^270^ ^]^ In addition, other compounds targeting the coagulation process could be helpful in inhibiting or preventing cerebral thrombosis.

Prevention of thrombin and thrombotic complications during CM and ECM can be achieved by blocking the procoagulation factor histidine‐rich protein II (HRPII),^[^ ^271^, ^272^ ^]^ administrating 2deoxyglucose ^[^ ^273^ ^]^ or convalescent plasma transfusion.^[^ ^196^ ^]^

Other broader immunomodulatory agents (e.g., modulators of TNF‐*α* production such as pentoxifylline, a phosphodiesterase inhibitor),^[^ ^233^ ^]^ oral activated charcoal (oAC) ^[^ ^274^ ^]^ and peroxisome proliferator‐activated receptor‐*γ* (PPAR‐*γ*) agonists ^[^ ^238^ ^]^ might help to preserve endothelial barrier integrity by suppressing the innate immune response that may injure the EC.^[^ ^275^ ^]^

All of these immunomodulators help prevent inflammation and ED and improve neuroprotection and upregulate the anti‐oxidant mechanisms in CM. Overall, the administration of classical anti‐parasite drugs and vaso‐protective treatments that protect EC dysfunction and BBB may significantly reduce mortality, decrease neuroinflammation, and neuronal damage and prevent cognitive impairment in CM.

Despite the administration of these various antimalarial therapies, the case fatality rate of CM remains extremely high because most of the drugs used do not substantially reduce morbidity or mortality.^[^ ^3^ ^]^ New therapeutic and mechanistic insights are constantly emerging from concerted biomedical research efforts which will hopefully allow us to identify safe and effective therapeutic targets and strategies. The development of new approaches to treat CM first and foremost requires a better understanding of the complex regulation of the cerebral microvasculature. The application of 2D and 3D models can be useful to thoroughly investigate CM and discover an appropriate therapeutic approach.

The emerging trends in drug delivery using nanobiotechnology have enabled a more effective and safe delivery of current anti‐malarial drugs and even a combination of them. This has revolutionized the therapeutic approach not only by improving patient compliance, cost effectiveness but also the ability to tailor them per the target requirement since it can enable a constant‐release of drug directly at the target location.^[^ ^276^, ^277^ ^]^ For example, Zhao et al have developed a ferritin nanozyme (Fenozyme) composed of recombinant human ferritin (HFn) protein shells that specifically target BBB ECs which has shown to significantly ameliorate the parasite induced BBB damage and improve the survivability of their experimental CM mouse model. This can be attributed to the inner Fe_3_O_4_ nanozyme core that displays ROS‐scavenging catalase‐like activity.^[^ ^278^ ^]^

## 5 Current Models to Investigate Cerebral Malaria

### 5.1 2D and 3D Co‐Cultures and Disease Models

The pathophysiology of CM involves sequestration of iRBC, immune cells, and endothelial activation, as evidenced by the co‐localization of IE on ICAM‐1‐positive in the brain microvascular system.^[^ ^16^, ^279^ ^]^ It has been suggested that the parasite has higher affinity for activated brain endothelium, but this concept has not been thoroughly investigated due to difficulties associated with accessing the brain, real‐time monitoring during various stages, and the lack of physiologically relevant models.^[^ ^87^, ^149^, ^280^ ^]^

Some of the main traditional approaches to investigate the transport across the brain endothelium include simple cell culture models of BMVECs, parallel artificial membrane permeability assays (PAMPA), transwell models, and in vivo models. While the latter are generally considered to be the most physiologically relevant because the brain endothelium is surrounded by its native microenvironment, animal models of CM lack relevance to human disease due to interspecies differences. In vivo models also have limited throughput. A major drawback of PAMPA is the over‐simplicity, as it cannot adequately represent the complexity and heterogeneity of the specialized BBB endothelium.^[^ ^281^ ^]^

Presently, mice and monkey models are considered the standard for studies on the BBB in ECM. Although these “in vivo” models have contributed significantly in elucidating the BBB mechanism, their utility is unfortunately limited by physiological differences between humans and animals, limiting reliable translation to clinical applications.

Conventional in vitro 2D modeling using human BBB ECs cultured in a flat static monolayer or co‐cultured with other glial cells offers a more “humanized” approach to address the translational limitation of animal models. Traditional Transwell systems with monolayers of BBB endothelium, or the more recent co‐culturing with other glial cells on either side of the insert, are one of the most commonly used systems for in vitro BBB models.^[^ ^282^ ^]^ They provide a more realistic platform for assessing BBB transport across the abluminal (brain‐facing) and luminal (bloodfacing) membranes, polarized expression and secretion, as well as real‐time evaluation of barrier integrity using transendothelial electrical resistance (TEER) measurements or tracer permeability, in addition to moderate high‐throughput scalability.^[^ ^281^, ^283^ ^]^ Despite the fact that Transwell and similar 2D models are relatively easy to use and are reproducible, they do not faithfully mimic the BBB microenvironment due to limitations in recapitulating complex cell–cell signaling and cell–ECM interactions due to the presence of the support membrane.^[^ ^284^ ^]^ Moreover, they lack some of the unique brain microvascular characteristics such as lumen shape and dimension, blood flow, and the resultant shear stress, which are known to be essential for the expression of the glycocalyx and key junctional and BBB genes and proteins.^[^ ^285^ ^]^

The complexity of the cellular network and interactions at the BBB and other blood vessels highlight the need for improved in vitro systems that better emulate organ‐specific blood vessels while incorporating time‐varying output that can accurately model physiological rates of blood flow. Recent advances in dynamic and 3D disease modeling including organoids and organ‐on‐chip technologies can provide more physiologically relevant platforms to study molecular mechanisms involved in malaria pathogenesis, advance our understanding of parasitevascular tropism in CM, reduce the use of animals and help the development of new treatment measures and therapies.^[^ ^45^ ^]^

Brain organoids have been developed to study the transport of brain‐penetrating molecules and organogenesis. They provide a system that can accurately model complex cell‐cell interactions by keeping cellular components in close juxtaposition in the absence of artificial membranes.^[^ ^286^ ^]^ These organoids are relatively easy to culture due to their small size, minimal culture requirements, and associated low maintenance costs, and are amenable to advanced imaging. This suggests that brain organoids can be suitable for studying parasite transport in response to various concentrations of different drugs in a semi high‐throughput format. Nevertheless, organoids have a number of major drawbacks, including the lack of controlled blood flow and hence a necroptotic core, inability to recapitulate physiological branching and curvature of blood vessels resulting endothelium‐lined spheres rather than vessels per se, limited capacity to incorporate multiple cell types without negatively impacting cellular viability and batch‐to‐batch variability.^[^ ^287^ ^]^

Different 3D perfusable micro‐ and macrovascular models with varying complexity have been engineered to better simulate the BBB microenvironment. Efforts to combine these 3D perfusable models with microfluidic systems resulted in the development of various “organ‐on‐a‐chip” models enabling study of the transmigration of pathogens, inflammatory mediators, the immune system, extravasation and metastasis of brain tumors and the successful formation of human brain microvessel‐like tubes of various shapes and diameters.^[^ ^288^, ^289^, ^290^ ^]^ Organ‐on‐a‐chip models are microphysiological systems that mimic the physiology and functionality of human organs. These models enable study of the mechanobiology of blood vessels in response to (patho)physiological shear stress and blood flow rates required for the activation of mechanosensing signaling pathways.^[^ ^285^ ^]^ Additional advantages of these organs‐on‐chips include, but are not limited to, the ability to measure the barrier integrity properties in real‐time, precise monitoring of cellular and structural dynamics, and biomarker detection following the interaction of iRBC with EC and extravasation using conventional and advanced imaging.^[^ ^93^, ^289^, ^291^ ^]^

The choice of cellular types can have a huge impact on the physiological relevance of organ‐on‐chip models.^[^ ^292^ ^]^ Animal cells are relatively easier to obtain and more widely available, but they have a limited physiological relevance simply due to interspecies differences with humans. Moreover, it has been shown that they have varied expression profiles of key blood vessel and BBB genes, including transporters and receptors, which further complicates the prediction of drug efficacy in humans.^[^ ^293^ ^]^

“Humanizing” in vitro models by using relevant cellular sources will greatly improve the translatability of results from these systems. Various immortalized lines of different BBB cellular components have been developed to address the translational limitation of using cells of animal origin. These cellular models represent an important tool for research, especially for drug screening using in vitro BBB models, as they have a number of advantages including limited batch‐to‐batch variability, relative ease‐of‐use, accessibility because they can be directly purchased from established sources, durability and longevity, because particular lines continue to retain some of the unique BBB characteristic required for biomedical studies despite being immortalized.^[^ ^294^ ^]^ However, one of the major limitations of immortalized cell lines is that they are leakier than cells of primary origin. They generally have reduced expression of tight junctions which are essential to produce an endothelium‐like feature of physiological BBB models.^[^ ^295^ ^]^ This may negatively impact their utility for the study of CM sequestration, parasite engulfment, and penetration through the BBB.

Primary human brain EC offers a better alternative to immortalized lines in modeling BBB physiology due to their high TEER values and lower permeabilities to tracers in different 2D and 3D BBB models. Although these cells are commercially available, they are often costly and have a relatively short lifespan. Moreover, primary cells can only be passaged a limited number of times and will stop dividing (or senesce) after a certain number of cell divisions, which significantly lowers their throughput.^[^ ^296^ ^]^ Primary human brain cells are generally derived from post‐mortem (including fetal) tissue or donors receiving resective temporal lobe surgery for refractory epilepsy. This results in limited availability, especially with recent surgical advances such as gamma knife, in addition to raising concerns regarding their reproducibility due to variability (including genetic variation) between donor individuals and translatability, as they are not obtained from healthy tissue.^[^ ^297^ ^]^

To avoid the reproducibility and availability limitations associated with the use of immortalized and primary cells, induced pluripotent stem cell (iPSC) models have recently been developed and promise to revolutionize the study of disease modeling of human disorders due to a number of advantages.^[^ ^298^ ^]^ These include their high proliferation and self‐renewal capacity, the ability to provide critical cell types which have been previously inaccessible or hard to obtain, the ability to create an unlimited (in principle) number of cells making them amenable for large‐scale production, stable genetics particularly associated with specific disorders such as BMECs from malaria patients and, excitingly, the possibility of isogenic BBB models having all cell types originating from the same donor.^[^ ^299^, ^300^ ^]^

Despite their high physiological relevance and advantages, there are a number of limitations associated with the use of iPSCs including the high cost of maintenance, the possibility of genetic variation due to epigenetic memory related to the reprogramming methodology, and variation in differentiation efficiency. The latter is an important aspect for modeling BBB for CM because the BMECs need a special differentiation protocol compared to any other EC (for example, peripheral EC) in order to ensure the presence of characteristic BBB properties. One of the most popular BMVEC differentiation protocols was published by Lippman et al. in 2012.^[^ ^301^ ^]^ Several improvements have been added to this protocol to ensure the proper phenotype of BMVECs and avoid generating a heterogenous mixture of epithelial/ ECs. The addition of retinoic acid, optimizing cell‐seeding density, balanced medium components, and the use of hypoxia have resulted in tighter EC (TEER values TEER 3000–4000 Ω.cm^2^), improved permeability, and higher expression of BMVEC markers such as ZO‐1, claudin‐5, PECAM‐1, occludin, glucose transporter type 1 (Glut‐1) and vascular endothelial (VE)‐cadherin.^[^ ^302^ ^]^ The use of these cells will result in more physiologically relevant BBB models to study the CM‐related endothelial activation and sequestration of *P. falciparum*.

In summary, 3D‐perfusable BBB‐on‐a‐chip using iPSC‐derived human cells represent excellent in vitro models to investigate the pathophysiology of CM and delineate the molecular mechanisms involved in penetrating the BBB endothelium. Future CM models can be further improved by the inclusion of other host blood cells, including platelets, rosettes, or neutrophils to enable investigation of the dynamic interaction between the parasite, immune factors, and inflammatory mediators at the site of BBB disruption and extravasation. These systems will benefit from having smaller diameters of artificial blood vessels and longer culture times for BMVECs to allow them to produce physiological extracellular matrix, which is known to be essential for proper BBB functioning and amenability for conventional and advanced live imaging to enable real‐time monitoring of BBB penetration and barrier function.^[^ ^289^, ^290^ ^]^ The systems will also have open designs to allow access to cells for further gene and protein expression profiling and also chemical fixation to enable further processing using transmission electron microscopy (TEM), high resolution volumetric imaging using Focused Ion Beam Scanning Electron Microscopy (FIB‐SEM), spatial transcriptomic using multiplexed error‐robust fluorescence in situ hybridization (MERFISH) and molecular histology using expansion microscopy.^[^ ^303^, ^304^, ^305^ ^]^

### 5.2 3D Bioprinting

Microfluidics have recently emerged as a promising alternative in the field of biomedical research because of the associated low operation and fabrication cost, high throughput, and amenability to automation. A major limitation of their widespread use is the complex and lengthy multi‐step lithographic processes. Recently, 3D bioprinting has become a new approach for the fabrication of microfluidics with physiologically‐relevant architectures using a wide range of materials while eliminating the need for lengthy and multi‐step processing.^[^ ^306^ ^]^

3D bioprinting is an advanced technology for microfluidic fabrication that can be implemented to build single or multicellular tissues that can emulate the 3D structure and geometry of real tissues. It has a number of advantages over traditional fabrication and molding techniques including the precise shape of channels, improved complexity and reproducibility, uniformity of microfluidic channels, lower cost and manufacturing time, and the ability to accurately print cells and structural scaffolds.

Recent advances in 3D bioprinting coupled with development of biomaterials that are compatible with existing printing techniques have enabled the successful handling of two or more biomaterials with suitable and compatible biological and mechanical characteristics to form mixed scaffolds that replicate the composition of native tissues while maintaining form fidelity,^[^ ^307^ ^]^ for example, using collagen I and IV together which are favored for brain EC and astroglia, respectively. This is essential for maintaining the tissue structure during cellular growth phase and hence the successful formation of physiologically‐relevant BBB models. The newly printed tissue models would enhance the reproducibility by minimizing the variability of cellular patterns from different constructs. This was evidenced by the recent work by Shöneberg et al. on vasculature‐on‐chip systems who bioprinted a 3D multi‐layer blood vessel that is indifferent from pipetting cells into the same hydrogel scaffold while maintaining around 80% cellular viability of human umbilical vein endothelial cells and smooth muscle cells.^[^ ^308^ ^]^

There are still some challenges for current 3D bioprinting approaches for blood vessels and BBB. These include (i) the biophysical characteristics of small liquid volumes which can limit the formation of small blood vessels with the necessary biomimetic nano‐resolution that is characteristic for BBB models, (ii) technical limitations in printing resolution that can affect the linear progression of blood vessel fabrication and (iii) validated iPSC differentiation protocols for the development of patient‐derived iPSCs to brain‐specific EC.^[^ ^307^ ^]^

However, we believe that the application of 3D bioprinting can be the future of in vitro investigation of CM pathogenesis. Usually, CM is investigated with model organisms such as mice or rats. Having the option to create blood vessels, the BBB, and other distinct tissues of the brain would allow investigation of the commencement of cytoadherence of iRBC and other milestones of CM development using a reductionist approach in a well‐defined experimental in vitro setup.

## 6 Conclusions

Malaria remains a major worldwide health concern due to its high morbidity and mortality. CM is one hallmark of disease severity and is nearly 100% fatal if untreated. Even with timely treatment, ≈15–20% of the patients do not survive CM. An infection with *P. falciparum* does not necessarily lead to this severe outcome, but an incidence of more than eleven thousand per million per year in the WHO African Region with the majority of patients being young children is unacceptably high.

Therefore, early detection of *P. falciparum* in the peripheral blood of the patient and subsequent diagnosis and treatment of CM is crucial to save the patient's life. Different detection methods and treatment options are available,^[^ ^309^ ^]^ but additional steps are needed to improve the outcome of CM. Vaccinations against CM are another option to protect people living in endemic areas. PfEMP1 variants that mediate cytoadherence in the microvasculature of the brain and even cause the breakdown of the BBB are particularly good vaccination targets.^[^ ^93^ ^]^ A recent study has further demonstrated that the administration of single‐doses of whole‐parasite from radiated‐attenuated sporozoites intravenously or subcutaneously offered protection from experimental CM in a rodent model.^[^ ^310^ ^]^ mRNA‐based vaccines are another possibility to achieve protection against CM, but the storage must be optimized to ensure usage in malaria endemic areas.^[^ ^311^ ^]^ Polymeric nanomaterials and nanocapsules are another potential CM vaccine alternative, but can also be applied as nanomedicines and in diagnostic approaches.^[^ ^312^ ^]^

The first step to ensure proper detection, treatment, and vaccine development is the thorough study of the development of CM. Many aspects of this disease are still shrouded in mystery. Therefore, the development of easily applicable 2D and 3D co‐cultures as well as disease models is necessary to decipher the pathophysiology of CM. Recent studies have provided valuable insights into how the parasite might disrupt BBB integrity using 3D spheroid models.^[^ ^93^ ^]^ Other in vitro bioengineering approaches include the use of 3D human brain‐specific microvessels to achieve a better understanding of how CM develops.^[^ ^313^ ^]^ Malaria tropica is a disease that can be controlled and prevented with the proper application of mosquito repellents and bed nets. However, an infection with *P. falciparum* is also not a death sentence. Malaria tropica is a curable disease and with the right diagnostics, treatment, and vaccination strategies, severe outcomes can and will be avoided. The groundbreaking approaches which are currently being developed by malaria research groups worldwide will make an impact on CM patients in the near future.

## Conflict of Interest

The authors declare no conflict of interest.
